# New restraints and validation approaches for nucleic acid structures in *PDB-REDO*

**DOI:** 10.1107/S2059798321007610

**Published:** 2021-08-24

**Authors:** Ida de Vries, Tim Kwakman, Xiang-Jun Lu, Maarten L. Hekkelman, Mandar Deshpande, Sameer Velankar, Anastassis Perrakis, Robbie P. Joosten

**Affiliations:** a Oncode Institute and Division of Biochemistry, Netherlands Cancer Institute, Plesmanlaan 121, 1066 CX Amsterdam, The Netherlands; b Department of Biological Sciences, Columbia University, New York, NY 10027, USA; c Protein Data Bank in Europe (PDBe), European Molecular Biology Laboratory, European Bioinformatics Institute (EMBL–EBI), Wellcome Genome Campus, Hinxton CB10 1SD, United Kingdom

**Keywords:** nucleic acid restraints, Watson–Crick base pairs, validation, *PDB-REDO*, *x*3*DNA-DSSR*

## Abstract

The quality of macromolecular structure models crucially depends on refinement and validation targets, which optimally describe the expected chemistry. Commonly used software for these two procedures has been designed and developed in a protein-centric manner, resulting in relatively few established features for the refinement and validation of nucleic acid-containing structure models. Here, new nucleic acid-specific approaches implemented in *PDB-REDO* are described, including a new restraint model using noncovalent geometries (base-pair hydrogen bonding and base-pair stacking) as refinement targets. New validation routines are also presented, including a metric for Watson–Crick base-pair geometry normality (*Z*_bpG_). Applying the *PDB-REDO* pipeline with the new restraint model to the whole Protein Data Bank (PDB) demonstrates an overall positive effect on the quality of nucleic acid-containing structure models. Finally, we discuss examples of improvements in the geometry of specific nucleic acid structures in the PDB. The new *PDB-REDO* models and pipeline are available at https://pdb-redo.eu/.

## Abstract 2

New restraints and validation targets for Watson–Crick base pairs are implemented in *PDB-REDO* and are applied to nucleic acid structures in the Protein Data Bank.

## 1. Introduction   

Refinement and validation are important steps in the process of obtaining reliable structure models of macromolecules from X-ray crystallographic experiments and cryo-electron microscopy (cryo-EM). Commonly used software during this process has been designed to be protein-centric and has resulted in many powerful tools for improving and optimizing protein models (Read *et al.*, 2011 ▸). Less emphasis has been placed on the optimization of nucleic acid structures, which has resulted in less established and integrated refinement and validation tools for nucleic acid-containing structure models. However, the number of nucleic acid-containing structure models in the Protein Data Bank (PDB; wwPDB Consortium, 2019 ▸) is increasing, notably also due to the successes achieved in resolving large protein–nucleic acid complexes using cryo-EM (Mitra, 2019 ▸). Therefore, the need for tools to validate and improve nucleic acid structure models becomes ever more pressing.

Parkinson and coworkers were the first to publish targets and tolerances for the covalent geometry of nucleic acid structures (Parkinson *et al.*, 1996 ▸). These were implemented in many refinement programs (Brünger, 1992 ▸; Blanc *et al.*, 2004 ▸; Sheldrick, 2008 ▸, 2015 ▸; Murshudov *et al.*, 2011 ▸; Liebschner *et al.*, 2019 ▸) and validation tools (Hooft *et al.*, 1996 ▸; Chen *et al.*, 2010 ▸; wwPDB Consortium, 2019 ▸). These targets have been updated several times, most recently by Kowiel, Gilski and coworkers, who defined updated targets for the bases, phosphates and (deoxy)riboses of nucleic acids separately (Kowiel *et al.*, 2016 ▸, 2020 ▸; Gilski *et al.*, 2019 ▸). Over the years, a large divergence in the used targets has arisen in the landscape of model refinement and validation software. This divergence often leads to conflicting details for users of refinement and validation software and for researchers analysing nucleic acid structure by data mining the PDB. To remedy this issue, the developers of these tools are working together to come to new consensus targets for nucleic acid covalent geometry (Schneider *et al.*, 2020 ▸). An important challenge in nucleic acid structure geometry optimization remains: the bond-length and bond-angle parameters for the (deoxy)riboses and the phosphate depend on the local conformation of the nucleic acid (Kowiel *et al.*, 2016 ▸, 2020 ▸).

**Figure 1:**
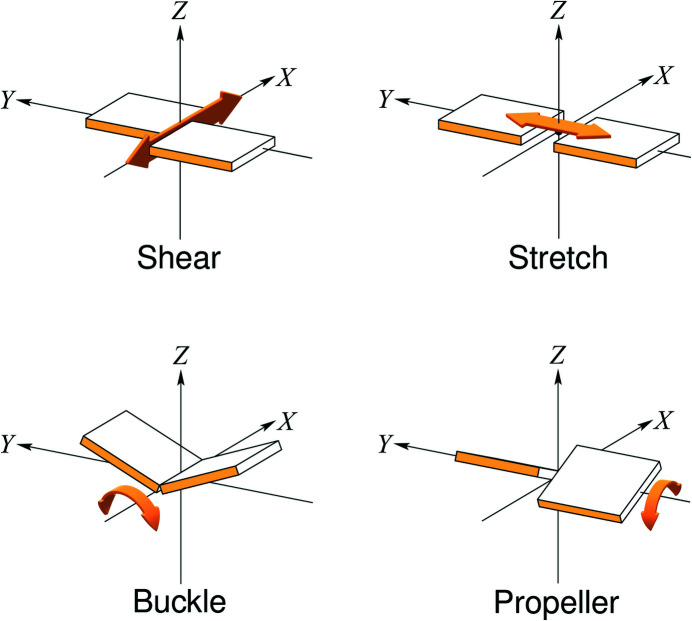
Simple parameters for nucleic acid structure analysis as described in *x*3*DNA-DSSR*.

Two validation tools are available for the conformations of nucleic acid structure models. Both check the conformations of sequential dinucleotides against a set of known conformers called ‘suites’ in *MolProbity* (Chen *et al.*, 2010 ▸) and ‘NtC classes’ in *DNATCO* (Černý, Božíková, Malý *et al.*, 2020 ▸). A notable difference is that *MolProbity* only checks RNA conformations based on the backbone torsion angles, whereas *DNATCO* also uses the relative orientation of the bases and checks both DNA and RNA conformations. Another difference is that *MolProbity* explicitly checks the sugar puckers of riboses in RNA and lists possible errors, whereas *DNATCO* reports on sugar puckers in DNA and RNA without explicit judgement of their quality. In terms of restraint generation a key tool is *LIBG* (Brown *et al.*, 2015 ▸), which can generate torsion restraints, base-stacking restraints and base-pair hydrogen-bond restraints. The *Phenix* suite (Liebschner *et al.*, 2019 ▸) also has nucleic acid restraints for base-pair hydrogen bonding, stacking and coplanarity of bases in a base pair. *BUSTER* (Blanc *et al.*, 2004 ▸) has specific restraints for ribose puckers. Despite the availability of restraint generators for base pairs in nucleic acids, no tools for the validation of base-pair geometry are available within the commonly used crystallographic refinement and validation packages. The most commonly used software for the analysis of nucleic acid structure is *x*3*DNA-DSSR* (Lu & Olson, 2003 ▸; Lu *et al.*, 2015 ▸; Li *et al.*, 2019 ▸). *x*3*DNA-DSSR* provides detailed information about hydrogen bonds, as well as descriptors for the spatial arrangement of base pairs. In addition to the well documented ‘local’ parameters (Lu & Olson, 2003 ▸), it also calculates a set of six ‘simple’ parameters (Li *et al.*, 2019 ▸), which provide a more intuitive interpretation of intra-base-pair structural variations, especially for the out-of-plane buckle and propeller distortions of noncanonical base pairs (Meier *et al.*, 2018 ▸). For the Watson–Crick base pairs that are the focus of this study, the local and simple parameters are virtually indistinguishable. To allow easy extension of the proposed pipeline to non­canonical (for example Hoogsteen) base pairs in the future, the four simple base-pair parameters shear, stretch, buckle and propeller (Fig. 1 ▸) were employed.

Here, we first implement the new covalent geometry targets for nucleic acids (Gilski *et al.*, 2019 ▸) and refine all of the high-resolution structures that contain nucleic acids in the PDB in *PDB-REDO* (Joosten *et al.*, 2012 ▸). We then use a high-quality subset of the resulting data set to generate new restraints based on hydrogen-bond targets for Watson–Crick (WC) base pairs. We implement all of the new restraints in a new *PDB-REDO* pipeline, which we applied to PDB entries that contain nucleic acids. We discuss specific examples where the new restraints correct modelling errors. The new PDB-REDO databank models, refined with a single protocol and consistent geometry targets, can provide a more reliable data set for users interested in specific structures and a better resource for data mining.

## 2. Experimental methods   

### 2.1. Data collection and data mining   

A local copy of the CCP4 Monomer Library (Vagin *et al.*, 2004 ▸; Nicholls *et al.*, 2021 ▸) was updated to incorporate the geometric targets for the standard DNA and RNA bases derived by Gilski *et al.* (2019 ▸). To ensure that all of the reference data were treated consistently and were of good quality, all nucleic acid-containing entries from the PDB-REDO databank (van Beusekom, Touw *et al.*, 2018 ▸) with diffraction data of resolution ≤2.0 Å were updated with *PDB-REDO* version 7.33 using the updated restraint files.

The resulting nucleic acid structure models were fed into *x*3*DNA-DSSR* to extract detailed information about the base pairs. From the output, the base pairs with their corresponding base-pair type, the hydrogen-bond distances between the two involved nucleotides and the ‘simple’ base-pair conformational parameters shear, stretch, buckle and propeller twist (Fig. 1 ▸) were extracted. A total of 1856 structure models (*i.e.* ‘redone’ PDB entries) were used to construct this data set.

In its default settings, *x*3*DNA-DSSR* is deliberately liberal in the detection of base pairs to avoid overlooking poorly modelled cases. This is an important feature when generating restraints for refinement, but can lead to poorer data sets in data mining. Therefore, in order to exclusively select high-quality data, only the base pairs for which both nucleotides had a real-space correlation coefficient (RSCC) of ≥0.95, as calculated by the program *density-fitness* (previously named *stats*; van Beusekom *et al.*, 2019 ▸), were retained. These pairs were divided into four categories based on the type of nucleotides involved: DNA–DNA base pairs, RNA–RNA base pairs, DNA–RNA base pairs and Other base pairs. Each of these categories was split into the base-pair types that *x*3*DNA-DSSR* distinguishes: Watson–Crick (WC), rWC, Wobble/~Wobble, rWobble, Sheared/~Sheared, Hoogsteen/~Hoogsteen, rHoogsteen/~rHoogsteen, Metal, Platform, Imino, Linker, Calcutta and Other (--); counts of the occurrences of all 13 base-pair types are shown in Supplementary Table S1. Based on the number of observations, only the WC base pairs were used in the determination of base-pair parameters (12 207 observations). Other base-pair types were observed 235 times or less, and therefore may be considered when more structural data become available.

### 2.2. WC base pairs: hydrogen-bond length targets   

The WC base-pair observations were split up further by the bases involved in the pair: types A–T, A–U and G–C. This brought the total number of different base pairs up to seven: two in DNA, two in RNA and three in DNA–RNA hybrids (Table 1 ▸). Base pairs containing modified nucleotides were kept separate. Consistent with the output from *x*3*DNA-DSSR*, modified nucleotides are marked with lowercase letters, for example a–u or G–c. All WC base pairs that did not contain the standard base-pair hydrogen bonds or for which *x*3*DNA-DSSR* could not determine simple parameters (for example because not all required atoms were present in the structure model) were discarded.

The counts, means and standard deviations of the hydrogen bonds observed in all seven base-pair types were then calculated. Two-sided *t*-tests at a confidence level of 99% (*p* < 0.01) were performed for the hydrogen-bond lengths in G–C base pairs in DNA and RNA to see whether these were significantly different (Supplementary Table S2). The test showed that the base-pair hydrogen bonds are significantly different; thus, the DNA–DNA and RNA–RNA G–C data could not be pooled. For completeness, two-sided *t*-tests comparing hydrogen bonds in DNA–RNA pairs with both DNA and RNA base pairs were performed as well (Supplementary Table S2). With the exception of O6–N4 hydrogen bonds in G–C base pairs and N6–O4 hydrogen bonds in A–U base pairs, the hydrogen bonds were significantly different between different types of nucleic acids, indicating that no other data could be pooled for the standard base pairs.

Base pairs with at least one modified nucleotide were investigated to see whether they could be treated as standard base pairs in the context of this study. The counts, means and standard deviations of the hydrogen bonds observed in modified nucleic acid base pairs were determined. The modified base pairs were compared with their corresponding natural base pair with a two-sided *t*-test at a confidence level of 99% (*p* < 0.01; Supplementary Table S3). In general, the modified base pairs do not significantly differ from their corresponding natural base pair in hydrogen-bond length. Therefore, the modified base pairs can be treated as natural base pairs. There were a total of 28 observations of A–U pairs in DNA and A–T pairs in RNA, some with modified nucleotides; these were removed from the data set because the number of observations was too small to derive additional targets.

To optimally define the target-mining data set, we also seek to define a resolution cutoff such that the resolution of the structures in the data set is as high as possible while still having sufficient observations to derive reliable targets. To this end, we performed the analysis above in steps of 0.05 Å from 2.00 to 1.50 Å. At 1.60 Å resolution, the DNA–DNA and RNA–RNA base pairs each had more than 300 observations (Table 1 ▸). At 1.55 Å resolution the number of observations decreased by 24%, which is a large loss of data for a small increase in coordinate precision. DNA–RNA hybrids are much more rare in structure models, which limits the number of observations. At 1.60 Å resolution the rarest base pair (A–U) has 43 observations, which is smaller than we would prefer, yet is similar to the number of observations previously used to derive geometric targets (see, for example, Parkinson *et al.*, 1996 ▸). Therefore, the cutoff was set to 1.60 Å. The means and standard deviations calculated for each hydrogen-bond type within the base pairs, 17 in total, are shown in Table 1 ▸. It is interesting to note that the mean hydrogen-bond distances in DNA–RNA hybrids are lower than their counterparts in DNA–DNA or RNA–RNA. The observed distributions of hydrogen-bond lengths are shown in Supplementary Fig. S1.

### 2.3. WC base pairs: simple parameters as validation targets   

The curated data set used to derive hydrogen-bond targets is also used to analyze geometric parameters for the relative base-plane orientations. For each of the seven base-pair types in the DNA–DNA, RNA–RNA and DNA–RNA categories, the counts, means and standard deviations of the simple parameters shear, stretch, buckle and propeller were determined. The sign of the shear and buckle values reported by *x*3*DNA-DSSR* depends on the base-pair direction (*i.e.* they are different for A–U and U–A pairs). This was taken into account during data mining by changing the sign of the obtained metric when needed. Two-sided *t*-tests at a confidence level of 99% (*p* < 0.01) comparing modified base pairs with their corresponding natural base pairs showed that the modified base pairs are not significantly different from the natural base pairs in most cases. This confirmed that the unnatural base pairs could also be treated as the natural base pair for the simple parameters. The means and standard deviations calculated at a 1.60 Å resolution cutoff are listed in Table 2 ▸ and the underlying distributions are shown in Supplementary Fig. S2. Among the four parameters, shear and stretch are around zero on average, as expected for WC pairs (Olson *et al.*, 2001 ▸). Shear consistently exhibits larger variations (by roughly twofold) than stretch. The mean value of propeller deviates significantly from zero, with A–T (A–U) pairs being more negative than G–C pairs. Buckle shows more pronounced fluctuations than propeller, except for A–T pairs in the DNA–RNA hybrid.

### 2.4. Testing nucleic acid restraint models   

The derived hydrogen-bond targets can be used as restraints in model refinement. To measure the effect of such restraints and to investigate the effect of using additional restraints for base stacking and backbone torsion angles, we defined the following six restraint models:

(i) Zero, with no additional nucleic acid restraints;
(ii) Mine, containing the mined hydrogen-bond restraints;
(iii) Stac, with the Mine restraints plus stacking restraints from *LIBG* (Brown *et al.*, 2015 ▸);
(iv) Tors, with the Mine restraints and torsion restraints from *LIBG*;
(v) Comb, combining all of the previous restraints;
(vi) LibG, with all targets derived from *LIBG*.

A data set of 6225 structure models, comprising the crystallographic PDB entries that contain nucleic acid base pairs and are available in the PDB-REDO databank, was refined in *REFMAC* (Murshudov *et al.*, 2011 ▸) with each of the six restraint models as so-called ‘external restraints’ using previously established refinement settings for geometric and *B*-factor restraint weights, *B*-factor models and solvent-mask parameters. These settings were taken from the PDB-REDO databank. Based on previous experience with hydrogen-bond restraints (van Beusekom, Touw *et al.*, 2018 ▸), the weight for the base-pair hydrogen-bond restraints was set to 2. Consistent with previous implementations in *PDB-REDO*, all restraints created with *LIBG* were used with the weight set to 5. The application of jelly-body restraints and the selection of the number of refinement cycles to ensure refinement convergence were based on the standard *PDB-REDO* algorithms for decision making (Joosten *et al.*, 2012 ▸). For all refined models, the following quality metrics were collected: *R*_free_, clashscore from *MolProbity* (Chen *et al.*, 2010 ▸), bond-angle root-mean-square *Z*-score (rmsZ) and rmsChiral from *REFMAC*, overall confal score from *DNATCO* (Schneider *et al.*, 2018 ▸; Černý, Božíková, Malý *et al.*, 2020 ▸), rmsZ for base-pair hydrogen bonds with respect to the targets derived in this study and rmsZ for all four simple parameters (shear, stretch, buckle and propeller; Li *et al.*, 2019 ▸). The structures were divided into five resolution bins each containing a similar number of models (0.55–1.90, 1.91–2.25, 2.26–2.64, 2.65–3.00 and 3.01–7.50 Å) and visualized as boxplots. Structures for which no *DNATCO* scores were obtained and severe outliers caused by technical issues (for exanple structures with largely overlapping alternate nucleic acid chains) were removed from the data set, leaving 6069 structures for analysis.

### 2.5. *PDB-REDO* implementation and high-throughput testing   

Analysis of the refinement results showed that the Stac restraint model was most suitable (see Section 3.2) for incorporation into *PDB-REDO*. The *PDB-REDO* software pipeline (Joosten *et al.*, 2012 ▸) can be divided into five stages: preparation, re-refinement, rebuilding, post-rebuilding refinement and finalization. It was adapted in the following ways.

(i) If the resolution of the diffraction data is worse than 1.70 Å, the re-refinement is now preceded by the generation of stacking restraints with *LIBG* and base-pair hydrogen-bond restraints with the program *bphbonds* (which uses *x*3*DNA-DSSR* for base-pair detection). The restraints are combined and used in *REFMAC* with weight 2 for hydrogen-bond restraints and weight 5 for stacking restraints. Residues with alternate conformations are excluded from these restraints.
(ii) At resolutions better than or equal to 1.70 Å no nucleic acid restraints are used, but *bphbonds* is used to generate target values for model validation.
(iii) A command-line option (--nonucrest) to switch off the use of nucleic acid restraints in *PDB-REDO* altogether was added.
(iv) The re-refinement stage is now followed by nucleic acid validation using three tools.
  (1) *Distel* is used to calculate the rmsZ score of hydrogen-bond length with respect to the restraints or targets set by *bphbonds*. It was updated from its previous version (van Beusekom, Touw *et al.*, 2018 ▸) to report the standard deviation based on a jackknife resampling estimate, similarly to as used for the Ramachandran plot *Z*-score (Sobolev *et al.*, 2020 ▸).
  (2) *Nucrmsz* is used to calculate the rmsZ scores and the estimated standard deviations (also based on the jackknife technique) for the simple base-pair parameters. *x*3*DNA-DSSR* is used within *nucrmsz* for structure analysis.
  (3) *Dnatco* is a local tool used to access *DNATCO* as a web service. The overall confal score and percentile are stored.
(v) The rebuilding stage of *PDB-REDO* is followed by recalculation of the restraints or hydrogen-bond target values to take into account changes in the structure model.
(vi) After the post-rebuilding refinement, the models are validated once more using *distel*, *nucrmsz* and *dnatco*.

To test the overall performance of the new *PDB-REDO* version, all PDB entries in the data set described in Section 2.4 were run though *PDB-REDO* twice, once with the default nucleic acid restraints and once with the --nonucrest option set (*i.e.* without nucleic acid restraints).

The resulting pairs of models were compared in terms of model-quality metrics (Figs. 3 and 5) and fitted to the electron density (Supplementary Fig. S5). *DNATCO* was used to analyze changes of sequential dinucleotide conformation in terms of CANA (Schneider *et al.*, 2018 ▸) class (Fig. 6).

### 2.6. Molecular graphics and data analysis   

Visual inspection of structure models was performed in *Coot* (Emsley *et al.*, 2010 ▸) and molecular-graphics figures were made in *CCP*4*mg* (McNicholas *et al.*, 2011 ▸). All plots were generated with the *seaborn* 0.11.1 plotting tool (Waskom, 2021 ▸) using *pandas* 1.1.2 (https://github.com/pandas-dev/pandas), Python 3.7.9 (van Rossum & de Boer, 1991 ▸) and *Matplotlib* 3.3.2 (Hunter, 2007 ▸) to import data and for optimization of the plots.

## 3. Results and discussion   

### 3.1. Validation and restraint targets   

The restraint and validation targets that we derived for hydrogen-bond lengths (Table 1 ▸) and the relative orientations of bases in WC base pairs (Table 2 ▸) differ significantly for different types of nucleic acids (DNA–DNA, RNA–RNA or DNA–RNA). However, there are no discernible differences between normal and modified nucleotides in base pairs. Alternative ways of splitting the data, such as splitting by secondary or tertiary structure (for example A-form, B-form, Z-form or other), as is used in protein structure validation (Sobolev *et al.*, 2020 ▸), are possible, but splitting by nucleic acid type has the clear advantage of covering all of the cases and being independent of the model quality. That is, the type of nucleic acid is known *a priori* with certainty, whereas the secondary or tertiary structures are not.

### 3.2. Comparing different restraint models   

**Figure 2:**
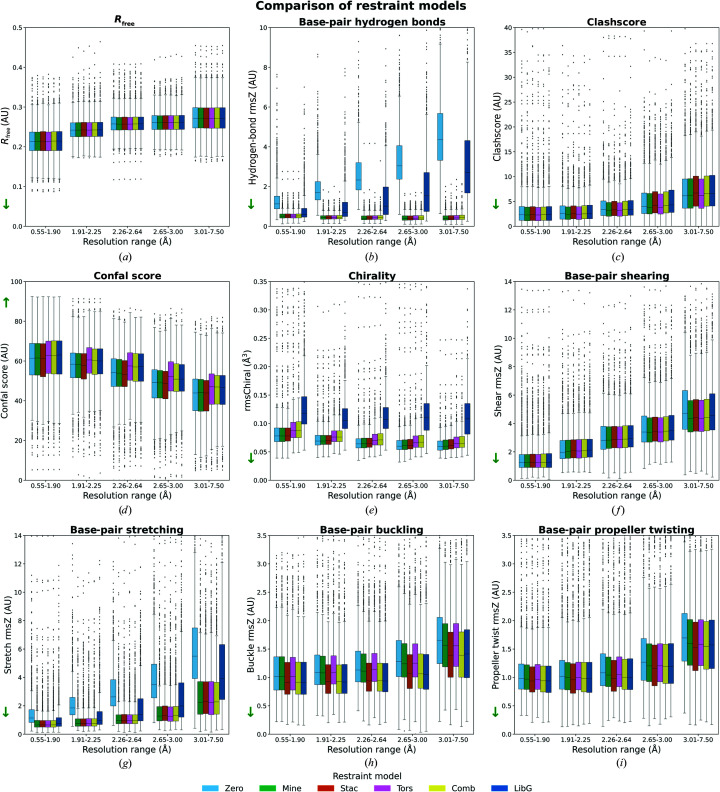
Comparison of the different restraint models Zero, Mine, Stac, Tors, Comb and LibG by (*a*) *R*_free_, (*b*) base-pair hydrogen bonds, (*c*) clashscore, (*d*) confal score, (*e*) chirality, (*f*) base-pair shearing, (*g*) base-pair stretching, (*h*) base-pair buckling and (*i*) base-pair propeller twisting. The *y* limits of the plots were adjusted for clarity. Boxplots containing all data can be found in Supplementary Fig. S4. The direction of better scores is indicated with an arrow on the *y* axis. AU stands for arbitrary units.

The new restraints were tested by comparing six different restraint models: Zero, Mine, Stac, Tors, Comb and LibG (Section 2.4). To compare these restraint models, general validation scores were calculated to analyse the differences independently. The *R*_free_ of the X-ray structure models is not affected substantially by any restraint model (Fig. 2 ▸ *a*), which is analogous to the effect of hydrogen-bond restraints in proteins (van Beusekom, Touw *et al.*, 2018 ▸). The effect of the restraints on base-pair hydrogen-bond rmsZ is, as expected, very large (Fig. 2 ▸ *b*). Without restraints (Zero), the rmsZ rapidly increases with worsening resolution. The LibG restraint model reduces the resolution effect but does not remove it. This is likely to be caused by *LIBG* using base-pair hydrogen-bond restraint targets that differ from those derived in this study, which are used as a reference for validation. The restraint models that use the hydrogen-bond length targets reported here remove the effect of resolution altogether. Other geometric parameters such as the bond-angle rmsZ are not affected by the choice of restraint model. The expected trend that the rmsZ decreases with worsening resolution is still observed (Joosten *et al.*, 2009 ▸; Supplementary Fig. S3). At this point we can conclude that the base-pair hydrogen-bond restraints are effective, but the choice of the most suitable restraint model is still unclear.

The *MolProbity* clashscore is a good metric for overall model quality, although in this study the effect of the restraints is diluted in protein–nucleic acid complexes because clashes within the protein are also considered in the score. The clashscore generally increases with worsening resolution (Fig. 2 ▸ *c*) and this trend is strengthened slightly by restraint models that include stacking restraints (Stac, Comb and LibG). This trend could be caused by the stacking restraints in *LIBG* becoming increasingly dominant at lower resolution. The interplanar distance target in the stacking restraints (3.4 Å) may not be ideal for all cases, making further optimization of stacking restraints a subject of further investigation. The confal score from *DNATCO* can be seen as an independent measure of model quality for nucleic acids, albeit focusing on sequential dinucleotides rather than base pairs. When comparing the different restraint models it is clear that the models that include torsional restraints (Tors, Comb and LibG) perform best (Fig. 2 ▸ *d*). However, these restraint models have an unfortunate side effect: they cause an increase in chiral volume restraint deviations, which are reported as rmsChiral by *REFMAC* (Fig. 2 ▸ *e*). The exact source of this effect is unclear. A likely cause is that the restraints are aimed at achieving specific ribose-pucker amplitudes which, for incorrectly built models, can only be reached by going through conformations with heavily penalized chiral volumes. A more suitable way of achieving correct ribose conformation would be real-space rebuilding rather than reciprocal-space refinement. Apart from this concern about chiral volume outliers, there is the more fundamental issue that the torsional restraints strongly optimize the values that *DNATCO* validates. This takes away the possibility of using the confal score as an independent validation metric. Using torsion restraint is the nucleic acid equivalent of using Ramachandran plot restraints in protein structure refinement and then saying that the Ramachandran plot has few or no outliers. It makes models look good at first sight, but can hide underlying pathologies (Sobolev *et al.*, 2020 ▸). Therefore, it was decided not to use restraint models that include torsion restraints in *PDB-REDO*.

The results above and related considerations reduce the restraint model options to just hydrogen-bond restraints (Mine) or hydrogen-bond plus stacking restraints (Stac). To make an informed choice, the simple base-pair parameters were analyzed. The rmsZ values corresponding to the shear and stretch parameters (Figs. 2 ▸ *f* and 2 ▸ *g*) show that no clear distinction could be made between the Mine and Stac restraint models. At low resolution the Mine restraint model is slightly better than the Stac restraint model, but the difference is too small to disqualify the latter model. In the plots representing the rmsZ of the buckle and propeller parameters (Figs. 2 ▸ *h* and 2 ▸ *i*) the Stac restraint model shows a substantially better performance compared with Mine, especially when it comes to base-pair buckling. Combining all of the results, the Stac restraint model was chosen for implementation into *PDB-REDO*.

A notable observation is that all rmsZ scores for the simple parameters (Figs. 2 ▸ *f*–2*i*) increase with worsening resolution. Base-pair shearing is affected most and there is already a clear difference between the highest and the second highest resolution set of models. Only in the lowest resolution set of models is there a clear effect of the use of any type of nucleic acid restraints. This suggests that base-pair shearing is an interesting candidate for further study.

### 3.3. High-throughput testing in *PDB-REDO*   

To test the performance of the Stac nucleic acid restraints in a practical setting, 6069 structure models from the PDB that contained WC base pairs were refined in the new version of *PDB-REDO*. This tests the new restraints combined with the geometric and *B*-factor restraint weighting procedures in *PDB-REDO*. As a control, the same calculations were performed without the nucleic acid restraints.

#### 3.3.1. Effect of *PDB-REDO* on nucleic acid and protein–nucleic acid complex structure models   

**Figure 3:**
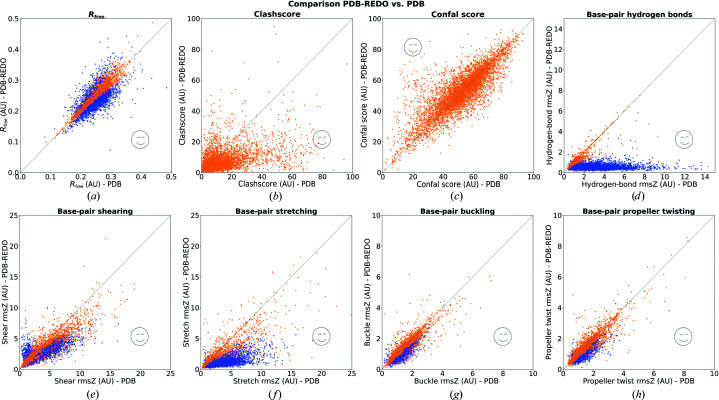
Diagonal plots of *PDB-REDO* versus PDB showing the effect on (*a*) *R*_free_, (*b*) clashscore (four outlier cases with clashscore > 100 in the PDB and one case with clashscore > 100 in *PDB-REDO* not shown), (*c*) confal score, (*d*) hydrogen-bond length rmsZ (one case with rmsZ = 20 in the PDB model not shown), (*e*) base-pair shearing, (*f*) base-pair stretching, (*g*) base-pair buckling and (*h*) base-pair propeller twisting. Emoticons mark the side of the diagonal that indicates an improvement. All scores are in arbitrary units (AU). Significant changes are marked in blue. No measure of significance is available for clashscore and confal score.

The current study marks the performance of *PDB-REDO* on nucleic acid structure models and complexes for the first time. In order to assess the significance of a change in a model-quality metric we use *Z*_change_, 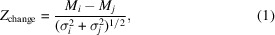
where *M* is the score for a specific metric and σ is the estimated standard deviation for that score. For *R*_free_ this is σ_*R*free_, as used routinely in *PDB-REDO* (Joosten *et al.*, 2014 ▸), and for the base-pair metrics the standard deviation was approximated based on jackknife resampling; for the clashscores and confal scores we were unable to estimate standard deviations. Fig. 3 ▸ shows comparisons of all eight model-quality indicators in the original PDB model and the *PDB-REDO* model. For the six metrics where we could estimate the standard deviation of the scores, significant changes (|*Z*_change_| > 2.6) are highlighted in blue. The confal score has the least clear improvement trend (Fig. 3 ▸ *c*). The clearest improvement is seen in the base-pair hydrogen-bond rmsZ as this is directly affected by the nucleic acid restraints. The cases where the clashscore (Fig. 3 ▸ *b*) and base-pair shearing rmsZ (Fig. 3 ▸ *e*) deteriorate cluster around the origin, *i.e.* where the original scores were already very good. An exception is one case where the clashscore increases from 72 to 110. In such cases the initial model requires more substantial model corrections than *PDB-REDO* can currently offer. It should be noted that 760 structure models had a data resolution of 1.70 Å or better. Those cases are refined without nucleic acid restraints to avoid biasing the PDB-REDO databank. Nevertheless, these cases are still relevant to assess the performance of *PDB-REDO* on nucleic acid structures and are still shown here.

**Figure 4:**
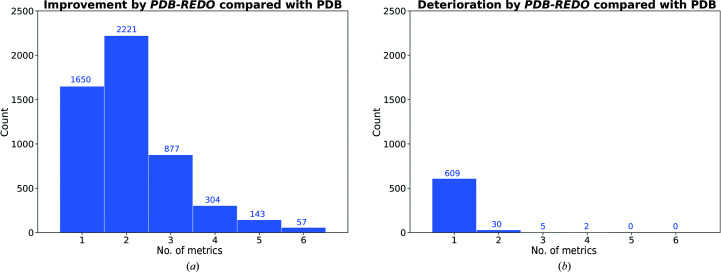
Distribution of the 6069 models in the test set in terms of the number of significantly (*Z* > 2.6) changed quality metrics (see Fig. 3 ▸) before and after *PDB-REDO*. The metrics considered are *R*_free_ and the base-pair rmsZ values. (*a*) Models improved by *PDB-REDO*, (*b*) models deteriorated by *PDB-REDO*.

Next, we wanted to examine the correlation between different metrics by indicating whether structure models that significantly improve (or deteriorate) in one model-quality indicator also become better (or worse) in other metrics (Fig. 4 ▸). About one in ten of all nucleic acid-containing structure models contain at least one significant change in the examined metrics. We observe that 5252 models (87% of all models) show significant improvement in at least one metric and 1381 of these (23%) show improvement in three metrics or more. In contrast, only 646 (11%) of the models show any significant deterioration in even one metric, while only seven show significant deterioration of three or more model-quality indicators. Overall, it can be stated that improvement in multiple structural aspects is much more common than deterioration in multiple structural aspects.

#### 3.3.2. Effect of *PDB-REDO* with versus without restraints   

**Figure 5:**
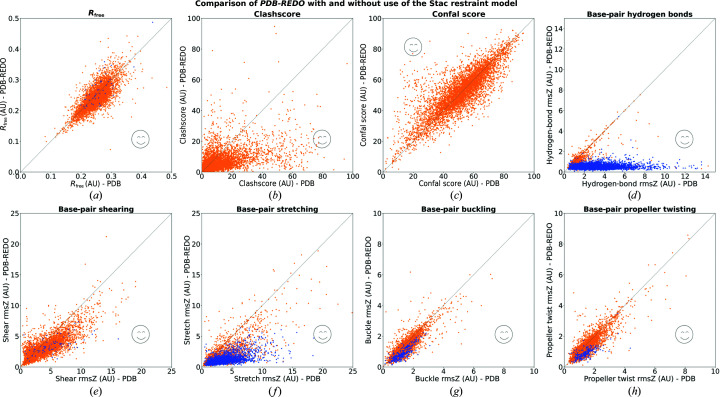
Diagonal plots of *PDB-REDO* with versus without restraints showing the effect on (*a*) *R*_free_, (*b*) clashscore (one outlier case with clashscore = 110 in both models not shown), (*c*) confal score, (*d*) hydrogen-bond length rmsZ, (*e*) base-pair shearing, (*f*) base-pair stretching, (*g*) base-pair buckling and (*h*) base-pair propeller twisting. Only structure models obtained with a resolution worse than 1.70 Å are shown. Emoticons mark the side of the diagonal that indicates an improvement. All scores are in arbitrary units (AU). Significant changes are marked in blue. No measure of significance is available for clashscore and confal score.

Besides showing the general effect that *PDB-REDO* has on model quality for nucleic acid structures and complexes deposited in the PDB, the specific effect of the Stac restraints model is investigated. Fig. 5 ▸ shows the comparison of models refined with and without Stac restraints using the same metrics as used in Fig. 3 ▸. In this comparison, models obtained with a resolution of 1.70 Å or better are excluded, as they are always refined without nucleic acid restraints.

The results are consistent with those of the restraint model selection (Section 3.2). The general scores *R*_free_ and clashscore show no obvious trend towards improvement or deterioration (Figs. 5 ▸ *a* and 5 ▸ *b*). The same is observed for the confal score (Fig. 5 ▸ *c*). As expected, the hydrogen-bond length rmsZ improves significantly in the *PDB-REDO* run with Stac restraints (Fig. 5 ▸ *d*). The rmsZ values for the simple parameters all decrease when the nucleic acid restraints are used but the number of significant changes varies greatly. The effect is most apparent in base-pair stretching, followed by buckling and propeller twisting. Base-pair shearing shows only a very limited significant effect (Figs. 5 ▸ *e*–5 ▸ *h*).

#### 3.3.3. Local model changes   

To analyze whether the conformational changes of base pairs brought on by the nucleic acid restraints also lead to a better fit to the electron-density maps, the real-space correlation coefficient (RSCC) for each nucleotide in the *PDB-REDO* models made with and without restraints was calculated with the program *density-fitness* (which was previously called *stats*; van Beusekom, Joosten *et al.*, 2018 ▸). Using the same procedure as used for the *PDB-REDO* server, the RSCC values were compared and a *Z*-score for the change in RSCC, *Z*_change_ (Joosten *et al.*, 2014 ▸), was calculated and plotted as a violin plot (Supplementary Fig. S5). As none of the absolute *Z*_change_ values exceeded the critical absolute value of 2.6, we conclude that the inclusion of nucleic acid restraints in refinement does not significantly affect the real-space density fit.

**Figure 6:**
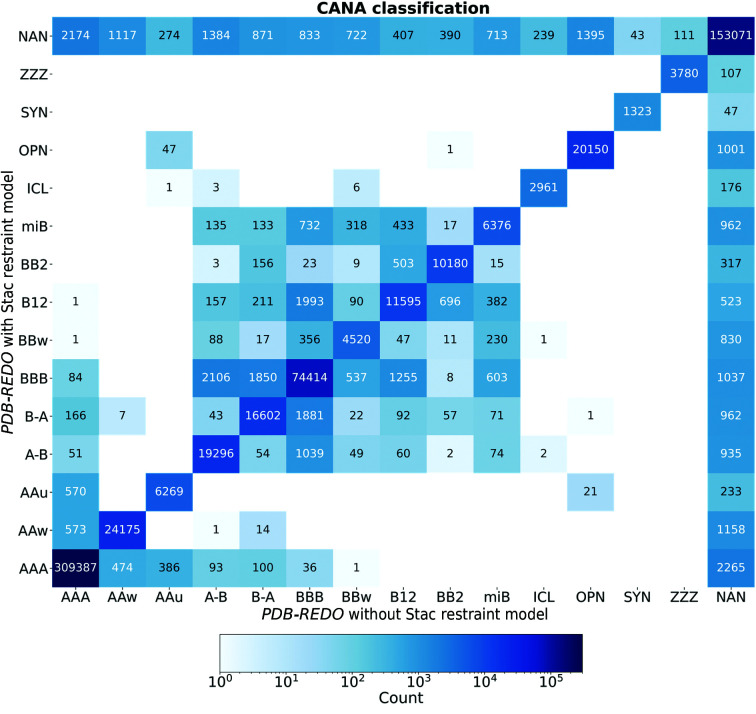
*DNATCO* CANA conformer classification of dinucleotides comparing the *PDB-REDO* models obtained using the Stac restraint model with the *PDB-REDO* models obtained without using the *Stac* restraint model. The number of observed changes is plotted in the cells, except for when zero changes were observed.

On the whole-structure scale, di­nucleotide geometry is not affected notably by the restraints in *PDB-REDO* judging by the confal score (Fig. 3 ▸ *c*), yet local changes may occur. To further examine this, the dinucleotide conformers as classified by *DNATCO* (Černý, Božíková, Svoboda *et al.*, 2020 ▸) in the *PDB-REDO* models refined with Stac restraints were compared with models refined without Stac restraints (Fig. 6 ▸). Additionally, both sets of models were compared with the conformers in the original PDB models (Supplementary Fig. S6). In terms of CANA classification, usage of the Stac restraint model caused very few changes in CANA class for dinucleotides apart from some shifts within the B-DNA classes. Also, compared with the original PDB models, most dinucleotides stay within their own class and movement is mostly restricted to classes associated with the same structural form; an exception is some movement within the A-DNA form. Using the more finely grained NtC classification does not change this overall trend. There is only movement into and away from nonclassified conformations (NAN); movement in either direction is of the same order of magnitude.

It should be noted that, as expected, the application of nucleic acid restraints from the Stac model has no clear effect on the movement between conformational classes (Fig. 6 ▸). Other restraint models that involve torsional restraint would likely behave differently as these also directly affect the confal scores (Fig. 2 ▸ *d*).

### 3.4. Example structures   

So far, only overall results for large sets of structure models have been shown. To illustrate the effect of *PDB-REDO* on nucleic acid structure models with individual structures, we decided to visualize the changes in structure as an overall base-pair geometry *Z*-score, *Z*_bpG_, defined and calculated for every base pair:
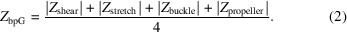
Base pairs with *Z*_bpG_ ≤ 3.00 are defined as normal, and higher values are considered to be outliers. It should be noted that a *Z*_bpG_ of >3.00 does not necessarily flag an incorrectly modelled base pair. The experimental data may support such a deviation from ideal geometry. The higher the *Z*_bpG_, the more likely that the base pair is modelled incorrectly.

#### 3.4.1. A reinterpretation of an RNA metal-binding site   

**Figure 7:**
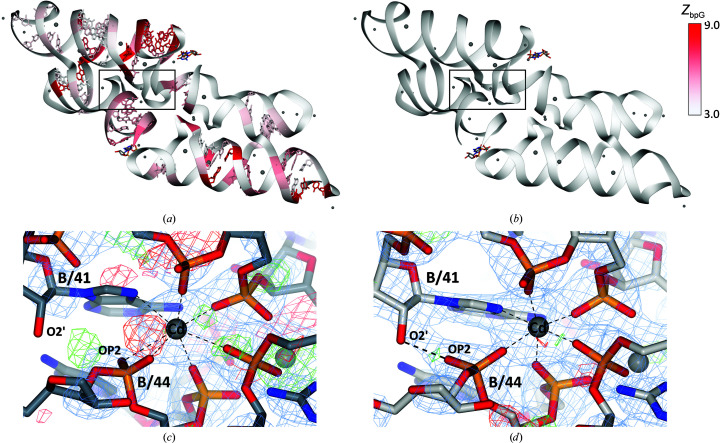
*YkoY*–*alx* riboswitch chimera bound to cadmium (PDB entry 6cc1) coloured by the mean *Z*-score of the simple parameters (*Z*_bpG_), illustrating the effects of the use of nucleic acid restraints in *PDB-REDO*. (*a*) Structure model as obtained from the PDB. Base-pair atoms are only displayed for pairs with *Z*_bpG_ > 3.0. (*b*) Structure model from *PDB-REDO*. Base-pair atoms are only displayed for pairs with *Z*_bpG_ > 3.0. (*c*) Binding site of B/203Cd in the PDB model. The ion is coordinated (thin dotted lines) by seven ligands including the OP1 and OP2 atoms of B/44A. 2*mF*_o_ − *DF*_c_ density map in blue at 2.0σ, *mF*_o_ − *DF*_c_ difference map in green and red at 3.0σ. For clarity, maps were oversampled with grid size 0.5. (*d*) The same binding site in the *PDB-REDO* model. The ion is now coordinated by six ligands as the OP2 atom of B/44A is reoriented towards O2′ of B/41A to form a hydrogen bond (thick dotted lines).

The *PDB-REDO*-ing of a structure model commonly leads to a higher quality structure model, but it can also change the (bio)chemical interpretation of the structure model (Touw *et al.*, 2016 ▸). This is for instance the case for the structure of the *ykoY*–*alx* riboswitch chimera bound to cadmium (PDB entry 6cc1, resolution 2.45 Å; Bachas & Ferré-D’Amaré, 2018 ▸). Overall, *PDB-REDO* improves *R*_free_ from 28.1% to 21.2% and the confal score from 53.2 to 56.1. The base-pair outliers in the original model (Fig. 7 ▸ *a*) are removed in the *PDB-REDO* model (Fig. 7 ▸ *b*). Sites that are not directly influenced by the nucleic acid restraints are also improved, especially the binding site of cadmium ion 203 in chain *B* (B/203Cd). In the original model the ion seems to have sevenfold coordination by, amongst others, both the OP1 and OP2 O atoms of B/44A (Fig. 7 ▸ *c*). This heptacoordination is a key point in the original study involving this structure. Strikingly, *PDB-REDO* changes this site to a normal sixfold coordination with a regular octahedral conformation without any explicit restraints towards this conformation. The phosphate group of B/44A reorients such that the OP1 O atom moves towards the ion and OP2 moves towards the ribose O2′ of B/41A, forming a hydrogen bond (Fig. 7 ▸ *d*). This change is further supported by the phosphate group of B/44A now adopting a better fit to the experimentally obtained density maps. Using the bond-valance parameters *R*_o_ = 1.875 and *B* = 0.37 for oxygen ligands and *R*_o_ = 1.951 and *B* = 0.37 for the N7 atom of B/41A (Palenik, 2006 ▸), the calculated bond valence (Brown & Altermatt, 1985 ▸) for B/203Cd is 0.76 in the original model and 2.26 in the *PDB-REDO* model. The latter is much closer to the expected value of 2.0 for a divalent cadmium ion. It is clear that the originally proposed heptacoordination involved in the ion specificity of this site (Bachas & Ferré-D’Amaré, 2018 ▸) is not supported by the crystallographic data.

#### 3.4.2. Large ribosomal structure improved by restraints and protein rebuilding   

**Figure 8:**
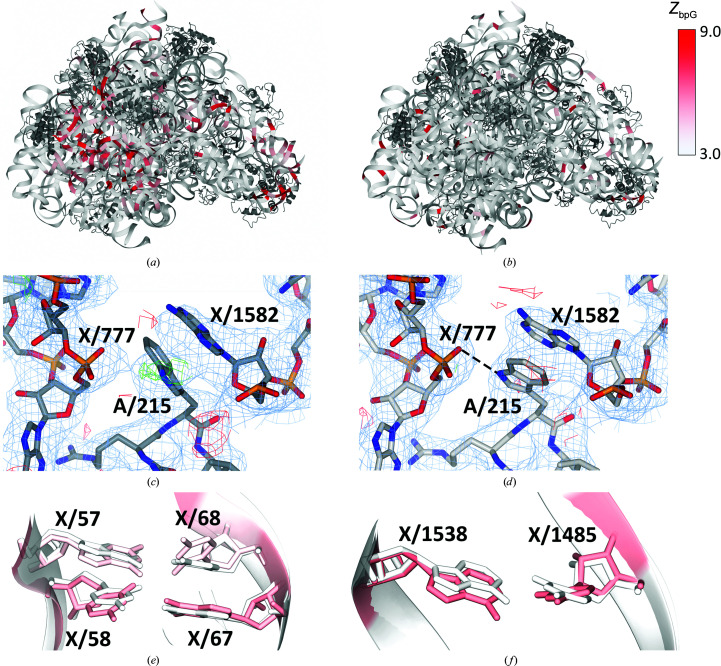
Ribosomal subunit (PDB entry 3pio) coloured by *Z*_bpG_, illustrating the effects of the Stac restraint model. Protein is coloured dark grey. (*a*) Structure model as available in the PDB. (*b*) Structure as obtained by *PDB-REDO*. (*c*) Protein–RNA interface site of A/215Trp and X/1582A. 2*mF*_o_ − *DF*_c_ density map in blue at 1.15σ, *mF*_o_ − *DF*_c_ difference map in green and red at 3.0σ. For clarity, maps were oversampled with grid size 0.5. (*d*) The same protein–RNA site in *PDB-REDO*, keeping the π-stacking but obtaining a better fit with the electron density and a hydrogen bond between the NE1 atom of A/215Trp and the OP2 atom of X/777A. (*e*) Overlay of the PDB model with the *PDB-REDO* model, both coloured by*Z*_bpG_, for base pairs X/57G–X/68C and X/58C–X/67G, which became more normal in the *PDB-REDO* model. (*f*) Overlay of the PDB with the *PDB-REDO* model, both coloured by *Z*_bpG_, of base pair X/1538A–X/1485U, which became more normal in the *PDB-REDO* model.

Another structure that illustrates the effectiveness of comprehensive model re-evaluation is the large ribosomal subunit of *Deinococcus radiodurans* in complex with lankamycin (PDB entry 3pio, resolution 3.25 Å; Belousoff *et al.*, 2011 ▸). For this model, neither *R*_free_ nor the confal score change noticeably, but the *Z*_bpG_ drops from 5.8 in the PDB model to 3.2 in the *PDB-REDO* model. The structure is modelled rather well in terms of base-pair normality (*Z*_mean_ ≤ 3.00) in the vicinity of the ligand, but worse *Z*_mean_ scores are obtained for the other parts of the ribosome (Fig. 8 ▸ *a*). Many outliers are removed by *PDB-REDO* (Fig. 8 ▸ *b*). An interesting structural change is found near A/215Trp in the 50S ribosomal protein L2. This residue is part of the protein–RNA interface and makes π interactions with the base of X/1582A. Model rebuilding by *SideAide* (Joosten *et al.*, 2011 ▸) flips the side chain of this tryptophan, thereby improving the RSCC of this residue from 0.79 to 0.88 and revealing a hydrogen bond between the NE1 atom of A/215Trp and the OP2 atom of X/777A (Figs. 8 ▸ *c* and 8 ▸ *d*).

The effect of the nucleic acid restraints in *PDB-REDO* is clearly seen by zooming in on base pair X/57G–X/68C (Fig. 8 ▸ *e*). The overall geometric adjustment, notably in terms of propeller twist, reveals the O6–N4 hydrogen bond (3.0 Å long) between the two nucleotides that is missing in the original model (interatomic distance of 3.9 Å). The *Z*_bpG_ score for this base pair improves from 4.28 to 0.69. The neighbouring base pair X/58C–X/67G is also improved by *PDB-REDO*. *Z*_bpG_ for this pair improves from 6.20 to 1.14, which is mainly brought on by repositioning X/58C. This nucleotide is twisted and is now positioned such that hydrogen-bond interactions can be formed (Fig. 8 ▸ *e*). The conformational changes of sequential residues are also reflected in a change of CANA class for the dinucleotide X/67G–X/68C, which goes from NAN to AAA. Perhaps somewhat surprisingly, the di­nucleotide X/57G–X/58C is classified as AAA in both the original and the *PDB-REDO* structure model.

Base pair X/1538A–X/1485U is improved by a large change in base-pair buckling. *Z*_bpG_ decreases from 7.12 to 1.83 in the *PDB-REDO* model (Fig. 8 ▸ *f*).

#### 3.4.3. Base-pair outliers near protein–DNA interfaces   

**Figure 9:**
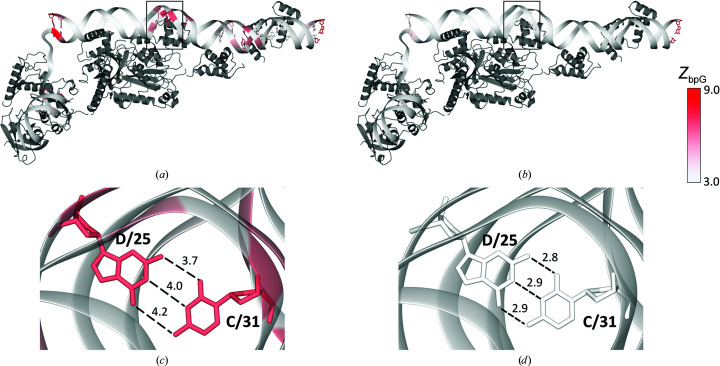
Protein–DNA complex of the bacteriophage Mu transposome (PDB entry 4fcy) coloured by *Z*_bpG_, illustrating the effects of the Stac restraint model. Protein is coloured dark grey. (*a*) Structure model as available in the PDB. Base-pair atoms are only displayed for pairs with *Z*_bpG_ > 3.0. (*b*) Structure as obtained by *PDB-REDO*. Base-pair atoms are only displayed for pairs with *Z*_bpG_ > 3.0. (*c*) Base pair C/31DC–D/25DG as deposited in the PDB with unlikely long base-pair distances (labelled in Å). (*d*) Base pair C/31DC–D/25DG from the *PDB-REDO* model with distances (labelled in Å) consistent with base-pair hydrogen bonding.

Geometric outliers either mark places where a model can be improved or genuine outliers that are of structural significance. However, regions of structural significance need not have geometric outliers. An example demonstrating this is a protein–DNA complex of the bacteriophage Mu transpososome (PDB entry 4fcy, resolution 3.71 Å; Montaño *et al.*, 2012 ▸). Most base pairs with *Z*_bpG_ above 3.0 (Fig. 9 ▸ *a*) are located near the protein–DNA interface, suggesting that these may be caused by local interactions. However, *PDB-REDO* optimization of this structure model using the Stac restraint model improves the *Z*_bpG_ for most of the base pairs and removes many outliers (Fig. 9 ▸ *b*). Apart from making the overall nucleic acid structure more normal, this shows that no distortions are caused by complex formation.

The value of the base-pair hydrogen-bond restraints is shown by the rmsZ for base-pair stretching. This is 4.19 for the PDB model but increases to 6.17 when the model is run through *PDB-REDO* without additional restraints. Employing the Stac restraint model allows *PDB-REDO* to reduce this to a much more likely value of 2.48. A clear example is seen in base pair C/31DC–D/25DG. The *Z*_bpG_ for this base pair in the initial structure was 6.0, while the model obtained from *PDB-REDO* using the new restraint model results in a *Z*_bpG_ of 0.5. More importantly, the bases were correctly oriented in the initial model but were too far apart to form good hydrogen bonds (N4–O6, 4.2 Å; N3–N1, 4.0 Å; O2–N2, 3.7 Å; Fig. 9 ▸ *c*). In the new structure model, the bases are positioned closer to each other as a result of using hydrogen-bond distance restraints, without changing the buckling or propeller twisting. The new base pair is conformationally normal, with strong hydrogen bonds (N4–O6, 2.9 Å; N3–N1, 2.9 Å; O2–N2, 2.8 Å; Fig. 9 ▸ *d*).

### 3.5. Availability and integration of validation metrics in the PDB-REDO databank and PDB   

The new restraint-generation and model-validation routines have been integrated into the *PDB-REDO* server, which is available at https://pdb-redo.eu. This server is freely available to users with an academic or commercial CCP4 (Winn *et al.*, 2011 ▸) licence. The updated structure models refined with the current *PDB-REDO* version are available through the PDB-REDO databank, which is also freely accessible at https://pdb-redo.eu. New or updated PDB entries are processed automatically when released by the wwPDB.

The PDB-REDO entry pages and user result pages were changed to show nucleic acid validation results in the ‘Model quality’ table, when applicable. RmsZ_bpG_ is shown as a percentile with respect to the PDB for the input and final structure models. Confal score percentiles from *DNATCO* are also shown. The underlying scores for both metrics are also available. Consistent with the other presented validation metrics, scores are highlighted in green when the percentile improves or in red when it deteriorates.

**Figure 10:**
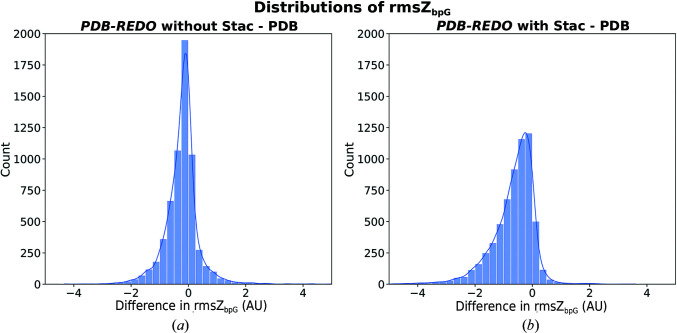
Distribution of base-pair normality changes after *PDB-REDO* model optimization (*a*) without and (*b*) with Stac restraints. Lower values are better.

The model-quality change sliders that are used on the user dashboard at https://pdb-redo.eu and on the PDBe entry pages at https://pdbe.org (Mir *et al.*, 2018 ▸) were also updated to show a slider for nucleic acid geometry. This slider is based on the change of rmsZ_bpG_. To define a cutoff for a meaningful change in rmsZ_bpG_, the distribution of changes compared with the PDB model was plotted for *PDB-REDO* models refined with and without nucleic acid restraints (Fig. 10 ▸). Without additional restraints (Fig. 10 ▸ *a*) there is a slight shift of rmsZ_bpG_ towards improvement; the peak is between −0.25 and 0. This is the shift that can be expected from just re-refinement. With restraints, a larger shift towards improvement is expected and indeed is observed (Fig. 10 ▸ *b*). We therefore consider a change in rmsZ_bpG_ of twice the ‘expected’ magnitude from just re-refinement to be meaningful, *i.e.* the difference in rmsZ_bpG_ is less than −0.5 or more than 0.5. Smaller changes at the level of a whole structure model are not considered to be significant and thus do not move the model-quality sliders away from the centre.

## 4. Conclusions   

To improve nucleic acid structure models, the base geometry restraints of Gilski *et al.* (2019 ▸) were implemented in *PDB-REDO*. Data mining for specific base-pair parameters allowed the testing of new restraint models which incorporated non­covalent restraints. These led to the Stac restraint model containing newly derived base-pair hydrogen-bond lengths and stacking restraints from *LIBG* for optimizing nucleic acid base-pair geometry.

The *Z*_bpG_ validation metric for Watson–Crick base-pair geometry normality was defined based on distributions of base-pair shearing, stretching, buckling and propeller twisting in high-quality structure models. The rmsZ_bpG_ over all WC base pairs in a model can be used as an overall quality indicator and local values can help in analysing structure models of nucleic acids to identify unusual geometries.

Based on high-throughput testing on 6069 PDB entries, we conclude that *PDB-REDO* with the new restraints improves nucleic acid structure models, as showcased for selected examples.

## Supplementary Material

Supplementary Tables and Figures. DOI: 10.1107/S2059798321007610/qn5003sup1.pdf

## Figures and Tables

**Table 1:** Target values for base-pair hydrogen bonds

Base-pair type	Base pair	Count	Hydrogen bond	Distance† (Å)	Standard deviation† (Å)
DNA–DNA	A–T	899	N1–N3	2.825	0.053
			N6–O4	2.999	0.099
	G–C	1544	O6–N4	2.901	0.095
			N1–N3	2.907	0.055
			N2–O2	2.830	0.078
RNA–RNA	A–U	301	N1–N3	2.829	0.051
			N6–O4	2.974	0.094
	G–C	675	O6–N4	2.916	0.088
			N1–N3	2.901	0.049
			N2–O2	2.819	0.070
DNA–RNA	A–T	92	N1–N3	2.800	0.029
			N6–O4	2.939	0.058
	G–C	131	O6–N4	2.889	0.051
			N1–N3	2.875	0.053
			N2–O2	2.779	0.098
	A–U	43	N1–N3	2.799	0.025
			N6–O4	2.963	0.039

† The precision used is for consistency with the typical precision used in the CCP4 Monomer Library.

**Table 2:** Target values for simple base-pair conformation parameters SD, standard deviation.

Base-pair type	Base pair	Count	Stretch (SD)† (Å)	Shear (SD)† (Å)	Propeller (SD)† (°)	Buckle (SD)† (°)
DNA–DNA	A–T	899	−0.111 (0.048)	0.042 (0.095)	−10.495 (6.315)	1.325 (7.327)
	G–C	1544	−0.130 (0.063)	−0.215 (0.101)	−6.432 (7.498)	−0.385 (8.453)
RNA–RNA	A–U	602	−0.103 (0.041)	0.042 (0.107)	−11.560 (4.560)	−0.720 (5.951)
	G–C	675	−0.131 (0.056)	−0.209 (0.115)	−10.979 (5.268)	−3.403 (5.437)
DNA–RNA	A–T	92	−0.135 (0.027)	0.103 (0.060)	−9.742 (3.033)	−5.269 (2.679)
	G–C	131	−0.150 (0.037)	−0.175 (0.078)	−7.512 (4.648)	0.838 (7.368)
	A–U	43	−0.133 (0.020)	0.007 (0.050)	−9.781 (2.039)	2.380 (2.356)

† The precision used is for consistency with the typical precision used in the CCP4 Monomer Library.
