# Supplementary material for: New restraints and validation approaches for nucleic acid structures in PDB-REDO
Source: Acta Crystallogr D Struct Biol. 2021 Aug 24;77(Pt 9):1127–41. doi: 10.1107/S2059798321007610 (PMC8411979; doi:10.1107/S2059798321007610)
Supplement: Supplementary file 1 [file d-77-01127-sup1.pdf]

STRUCTURAL
BIOLOGY

Volume 77 (2021)

Supporting information for article:

New restraints and validation approaches for nucleic acid structures in *PDB-REDO*

Ida de Vries, Tim Kwakman, Xiang-Jun Lu, Maarten L. Hekkelman, Mandar Deshpande, Sameer Velankar, Anastassis Perrakis and Robbie P. Joosten

Table S1 Observed base pairs in PDB-REDO at 2.00 Å resolution cut-off and RSCC ≥ 0.95.

Base pair type	All base pairs	DNA-DNA base pairs	RNA-RNA base pairs	DNA-RNA base pairs	Other base pairs
WC	12207	8100	2847	653	607
Wobble	235	16	209	2	8
Sheared	166	24	139	0	3
Metal	112	96	15	0	1
rHoogsteen	115	9	96	0	10
Platform	83	13	64	1	5
Imino	44	7	37	0	0
rWC	30	9	10	0	11
Linker	41	41	0	0	0
Hoogsteen	27	10	14	0	3
Calcutta	22	2	20	0	0
rWobble	1	0	0	0	0
Other (--)	4030	1939	1770	41	280

Table S2 *t*-tests of Watson-Crick (WC) base pairs at 1.60 Å resolution cut-off.

T-test comparison	Base pair	Hydrogen bond	*t*-value*	Critical *t*-value#
DNA vs. RNA	G-C	O6-N4	**3.517**	2.626
		N1-N3	**2.800**	2.626
		N2-O2	**3.258**	2.626
DNA-RNA vs. DNA	A-T	N1-N3	**7.109**	2.639
		N6-O4	**8.643**	2.639
	G-C	O6-N4	2.371	2.626
		N1-N3	**6.612**	2.626
		N2-O2	**5.762**	2.626
DNA-RNA vs. RNA	A-U	N1-N3	**6.160**	2.704
		N6-O4	1.390	2.704
	G-C	O6-N4	**4.767**	2.626
		N1-N3	**5.064**	2.626
		N2-O2	**4.423**	2.626

* Bold numbers indicate that compared hydrogen bond distances differ significantly

# Defined at confidence level of 99% ($p < 0.01$).

Table S3 Unnatural WC base pairs at 1.60 Å resolution cut-off.

Base pair type	Base pair	Count	Hydrogen bond	Distance (Å)*	Standard* deviation (Å)	*t*-value (critical *t*) #
DNA-DNA	a-t	13	N1-N3	2.761	0.021	**10.426 (3.055)**
			N6-O4	2.975	0.031	2.611 (3.055)
	g-c	0	O6-N4			
			N1-N3			
			N2-O2			
RNA-RNA	a-u	20	N1-N3	2.808	0.042	2.129 (2.861)
			N6-O4	2.985	0.069	0.664 (2.861)
	g-c	34	O6-N4	2.860	0.046	**6.492 (2.750)**
			N1-N3	2.893	0.044	0.950 (2.750)
			N2-O2	2.834	0.058	1.416 (2.750)
DNA-RNA	a-t	21	N1-N3	2.800	0.029	1.529 (2.845)
			N6-O4	2.939	0.058	**6.789 (2.845)**
	c-g	25	O6-N4	2.889	0.051	**4.379 (2.797)**
			N1-N3	2.875	0.053	2.274 (2.797)
			N2-O2	2.779	0.098	**2.920 (2.797)**
	a-u	27	N1-N3	2.799	0.025	0.392 (2.779)
			N6-O4	2.963	0.039	2.250 (2.779)

* The used precision is for consistency with the typical precision used in the CCP4 Monomer Library.

# Compared to corresponding natural hydrogen bond distance, bold numbers indicate that unnatural base pair differs significantly from the corresponding natural base pair at confidence level of 99% ($p < 0.01$).

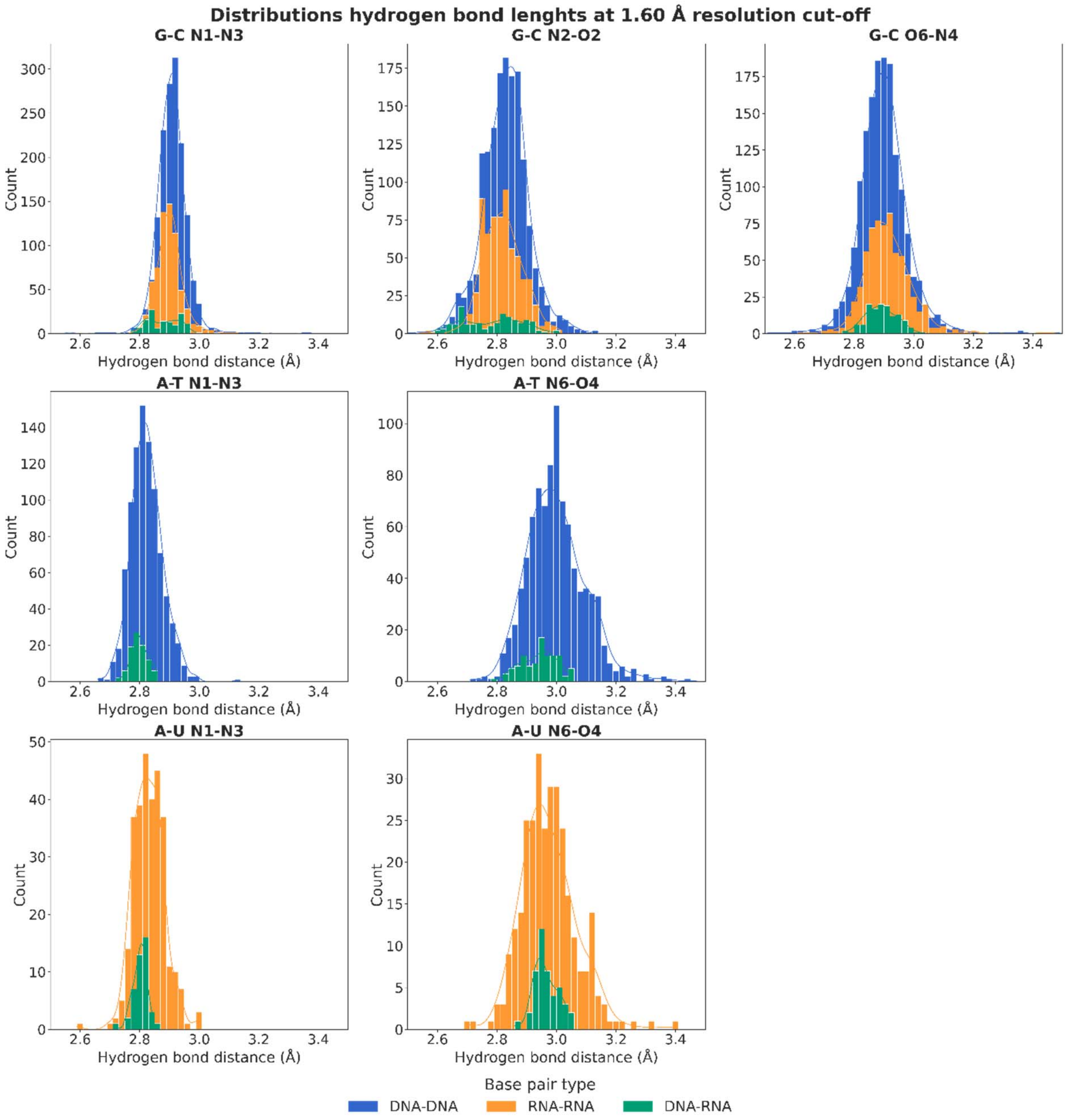

Figure S1 Distributions of the observed hydrogen bond length values, plotted with a 0.02 Å bin width.

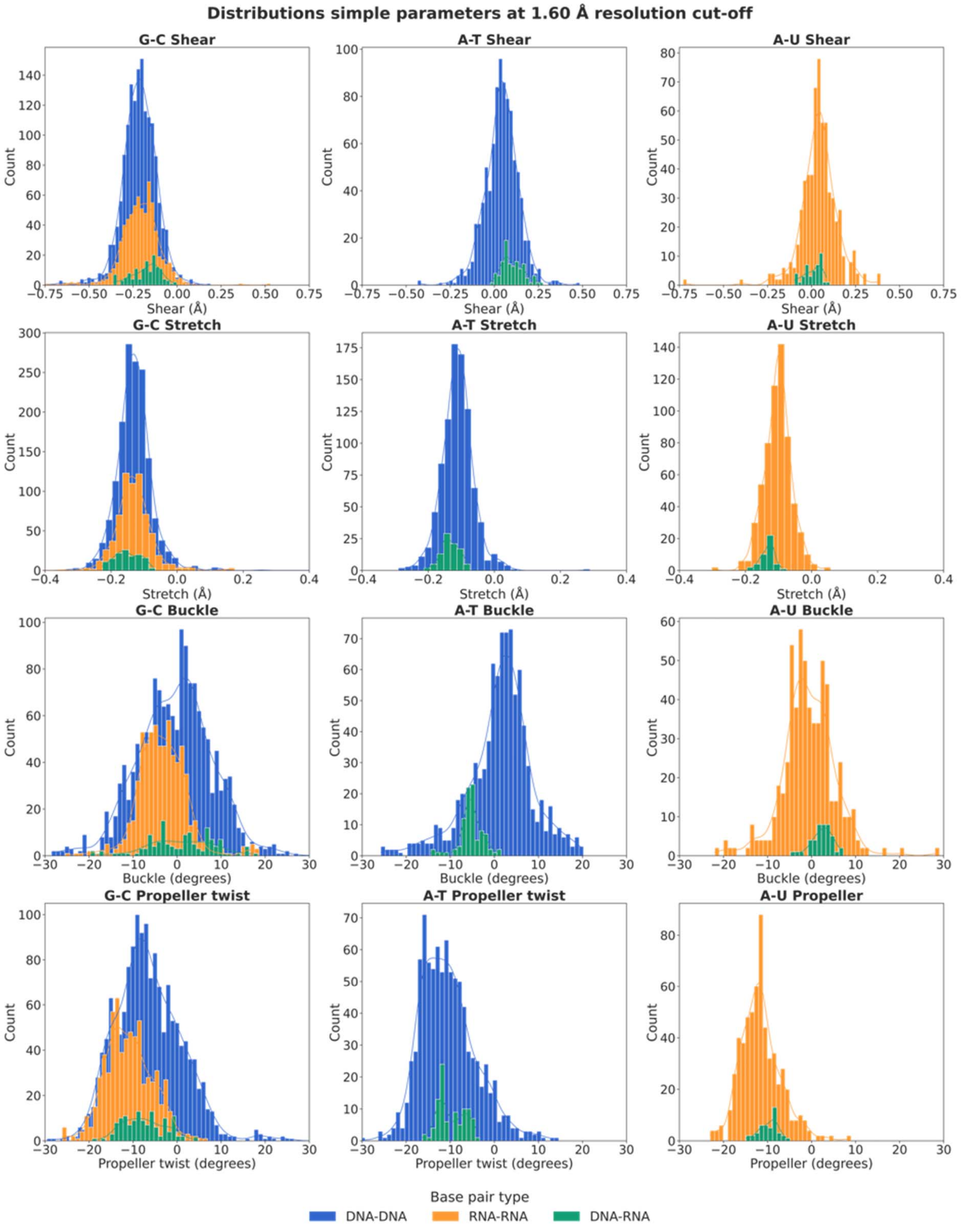

Figure S2 Distributions of the observed simple base-pair parameter values, plotted with a 0.02 Å bin width for shear and stretch, and a 1.0° bin width for buckle and propeller twist.

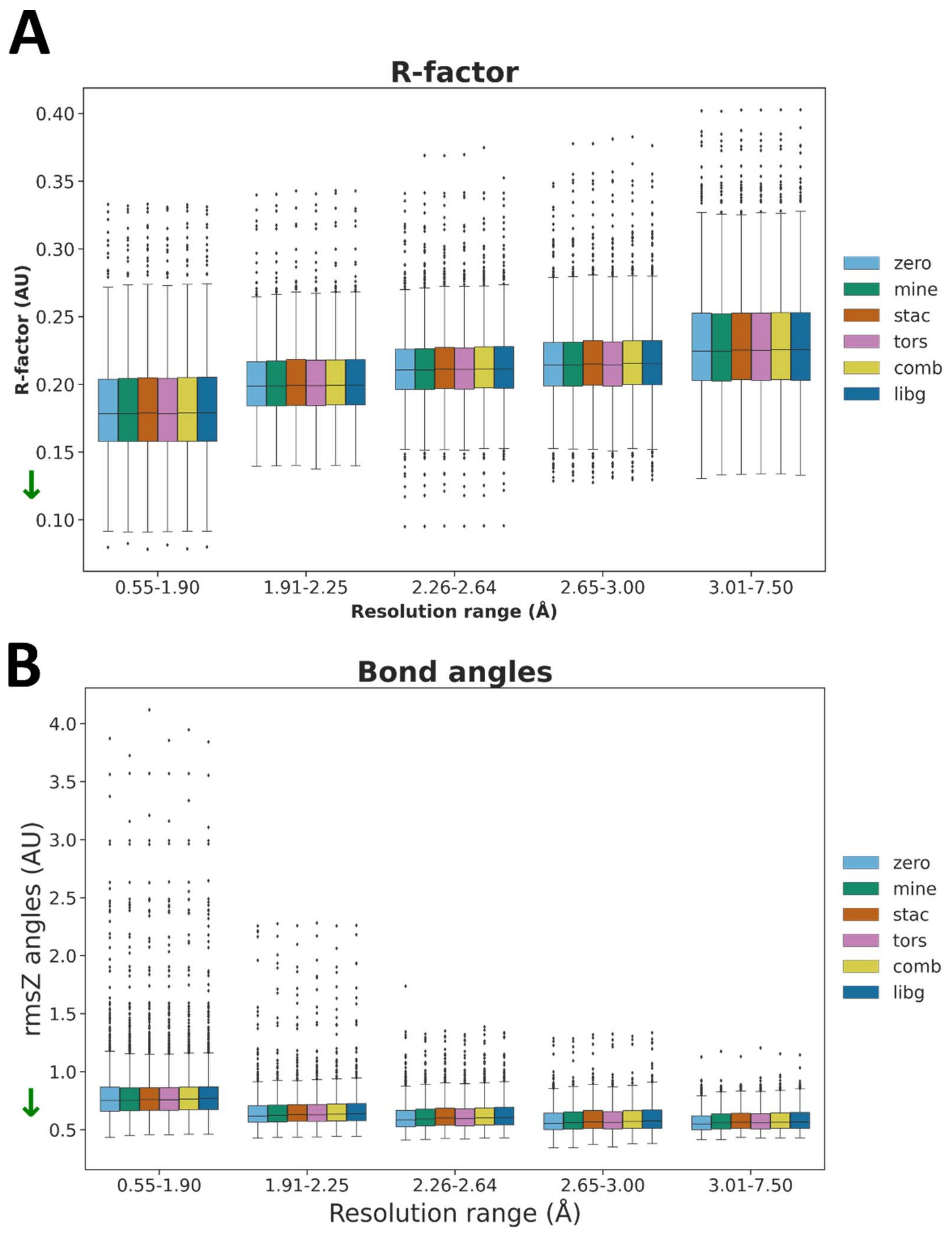

Figure S3 Boxplot of A) R-factor, B) the bond angle rmsZ as reported by *REFMAC*, in testing different restraint models. The direction of better scores is indicated with an arrow on the y-axis. AU stands for arbitrary units.

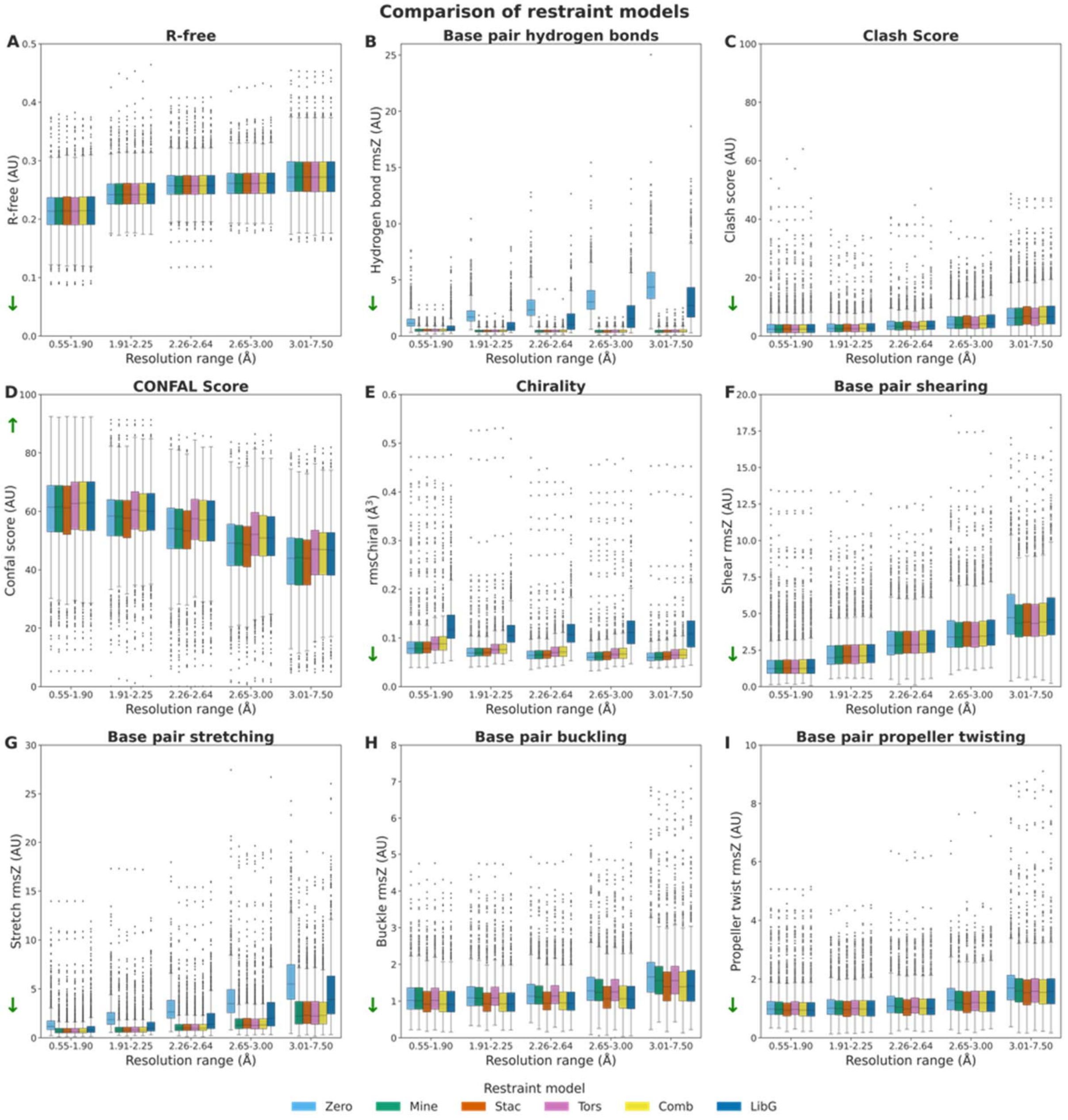

Figure S4 Comparison of different restraint models Zero, Mine, Stac, Tors, Comb and *LIBG* (all observations): A) R-free, B) Base pair hydrogen bonds, C) Clash Score, D) CONFAL Score, E) Chirality, F) Base pair shearing, G) Base pair stretching, H) Base pair buckling, I) Base pair propeller twisting. The direction of better scores is indicated with an arrow on the y-axis. AU stands for arbitrary units (AU).

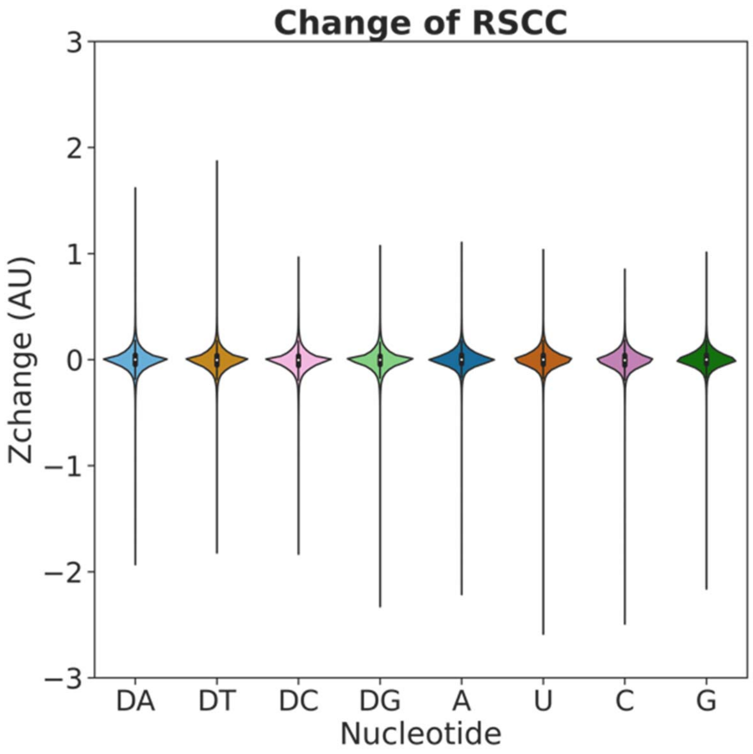

Figure S5 Violin plot expressing the change in RSCC by Z_{change} calculated after using nucleic acid restraints in refinement with *PDB-REDO*. AU stands for arbitrary units.

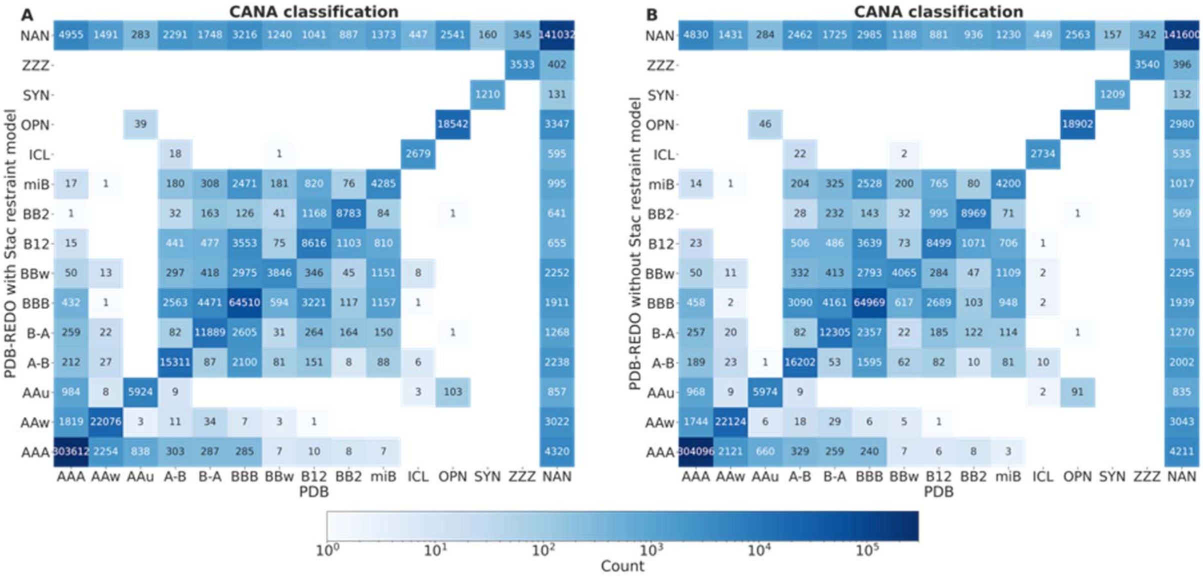

Figure S6 *DNATCO* CANA conformer classification of dinucleotides comparing the original PDB structure models to A) PDB-REDO models obtained using the Stac restraint model, B) PDB-REDO models obtained without using the Stac restraint model.
